# Overexpression of a *Metallothionein 2A* Gene from Date Palm Confers Abiotic Stress Tolerance to Yeast and *Arabidopsis thaliana*

**DOI:** 10.3390/ijms20122871

**Published:** 2019-06-12

**Authors:** Himanshu V. Patankar, Ibtisam Al-Harrasi, Latifa Al Kharusi, Gerry Aplang Jana, Rashid Al-Yahyai, Ramanjulu Sunkar, Mahmoud W. Yaish

**Affiliations:** 1 Department of Biology, College of Sciences, Sultan Qaboos University, P.O. Box 36, 123 Muscat, Oman; himanshu30@gmail.com (H.V.P.); i.alharrasi@gmail.com (I.A.-H.); latifakharusi@gmail.com (L.A.K.); gerryjana@gmail.com (G.A.J.); 2 Department of Crop Sciences, College of Agricultural and Marine Sciences, Sultan Qaboos University, P.O. Box 34, 123 Muscat, Oman; alyahyai@squ.edu.om; 3 Department of Biochemistry and Molecular Biology, Oklahoma State University, Stillwater, OK 74078, USA; ramanjulu.sunkar@okstate.edu

**Keywords:** metallothionein, abiotic stress, date palm, salinity, drought, functional characterization

## Abstract

Although the date palm tree is an extremophile with tolerance to drought and certain levels of salinity, the damage caused by extreme salt concentrations in the soil, has created a need to explore stress-responsive traits and decode their mechanisms. Metallothioneins (MTs) are low-molecular-weight cysteine-rich proteins that are known to play a role in decreasing oxidative damage during abiotic stress conditions. Our previous study identified date palm metallothionein 2A (*PdMT2A*) as a salt-responsive gene, which has been functionally characterized in yeast and *Arabidopsis* in this study. The recombinant PdMT2A protein produced in *Escherichia coli* showed high reactivity against the substrate 5′-dithiobis-2-nitrobenzoic acid (DTNB), implying that the protein has the property of scavenging reactive oxygen species (ROS). Heterologous overexpression of *PdMT2A* in yeast (*Saccharomyces cerevisiae*) conferred tolerance to drought, salinity and oxidative stresses. The *PdMT2A* gene was also overexpressed in *Arabidopsis*, to assess its stress protective function in planta. Compared to the wild-type control, the transgenic plants accumulated less Na^+^ and maintained a high K^+^/Na^+^ ratio, which could be attributed to the regulatory role of the transgene on transporters such as HKT, as demonstrated by qPCR assay. In addition, transgenic lines exhibited higher chlorophyll content, higher superoxide dismutase (SOD) activity and improved scavenging ability for reactive oxygen species (ROS), coupled with a better survival rate during salt stress conditions. Similarly, the transgenic plants also displayed better drought and oxidative stress tolerance. Collectively, both in vitro and in planta studies revealed a role for *PdMT2A* in salt, drought, and oxidative stress tolerance.

## 1. Introduction

Abiotic stresses are rapidly affecting agricultural lands, constantly diminishing the productivity and quality of agricultural crops [1,2]. The global population is escalating and the demand for food production is rapidly increasing [3]. In order to provide a solution for sustainable agriculture, improving the ability of plants to tolerate abiotic stresses is necessary [4]. Date palm (*Phoenix dactylifera* L.) is an economically important fruit tree and has a long history of cultivation in the Middle East and Northern Africa [5]. Date palm cultivation is a major component of Oman’s agriculture and the country is amongst the top 10 date palm producers in the world [6]. In recent years, date palm cultivation has been markedly affected by invading abiotic stresses, including salinity, and therefore improving the plant’s stress tolerance is a necessity [7,8]. Overuse of groundwater reserves, lack of rainfall and irrigation with brackish water are the main causes of drought and salinity in this region [9,10].

The date palm plant is an extremophile with tolerance to various abiotic stresses such as heat, drought and salinity; however, lately the productivity and quality of fruits has been affected by these stress conditions [7,11]. Studies on the response of date palm to abiotic stresses are limited and the explicit mechanisms of stress tolerance are yet to be identified. The transcriptome, methylome and miRNAome of salinity-stressed date palm offer some insights into the complex abiotic stress tolerance mechanisms [12,13,14,15,16,17]. Specifically, the global gene expression profiles of salt-stressed date palm have identified a differentially expressed genetic makeup [12]. Along the same lines, a yeast functional bioassay aimed at identifying salt-stress-responsive genes has identified several genes, including a metallothionein (MT) gene [18]. 

MTs are low-molecular-weight cysteine-rich proteins, well known for their role in the sequestration of heavy metals such as cadmium and mercury. In addition, MTs play an important role in regulation of homeostasis of essential metals such as zinc and copper [19,20]. In addition to sequestration of heavy metals, the cysteine residues of the MTs are directly and indirectly involved in the elimination of reactive oxygen species (ROS), which tend to accumulate to toxic levels during abiotic and biotic stress conditions [21,22,23,24,25]. MTs are present in all plants, animals and fungi and also in some prokaryotic organisms [19]. MTs are classified into 15 families, based on their taxonomic features and the distribution of the cysteine residues [20]. MTs in plants belong to family 15 and are further divided into four types (i.e., MT1 (12 Cys), MT2 (14 Cys), MT3 (10 Cys) and MT4 (17 Cys) [21]). Several studies have previously shown that MTs play a role in conditions of drought and salinity [24,26,27,28,29,30]. In addition, the expression of MTs is regulated by various factors, including environmental stimuli, pathogens, wounds, cold, heat stress and hormone treatment [31,32]. For instance, transgenic tobacco overexpressing the *GhMT3a* gene of *Gossypium hirsutum* L., displayed improved tolerance of cold, drought and salinity. Similarly, transgenic *Arabidopsis* expressing the *ZjMT* gene of *Ziziphus jujuba* exhibited enhanced salinity tolerance during germination [33,34]. Additionally, MTs are important players in a variety of physiological processes, such as seed germination, root development and fruit ripening [35,36,37]. Furthermore, MTs are widely considered to be a component of the antioxidant defense system [38] and could also be involved in the modulation of the basic transcription process by regulating the binding to DNA of zinc finger peptides [39].

In this study, an attempt was made to functionally characterize date palm’s salt-stress-inducible metallothionein 2A (*PdMT2A*) gene in yeast and transgenic *Arabidopsis thaliana.* The aim of the study was to determine the ability of *PdMT2A* to enhance abiotic stress tolerance, especially under drought and salinity stress conditions. The results revealed that the transgenic yeast strain was more tolerant to drought, salinity and oxidative stresses. Similarly, the transgenic Arabidopsis plants overexpressing *PdMT2A* exhibited improved tolerance when exposed to drought, salinity and oxidative stress conditions. The results obtained from this study may provide valuable information relating to the abiotic stress tolerance mechanisms in date palm. Determination of the function of genes involved in common abiotic tolerance mechanisms, such as MTs, is a prerequisite for identification of novel mechanisms in date palm, as a model abiotic-stress-tolerant fruit plant.

## 2. Results

### 2.1. Sequence Analysis Revealed the Presence of Common Metallothionein Fingerprints for PdMT2A

**Figure 1:**
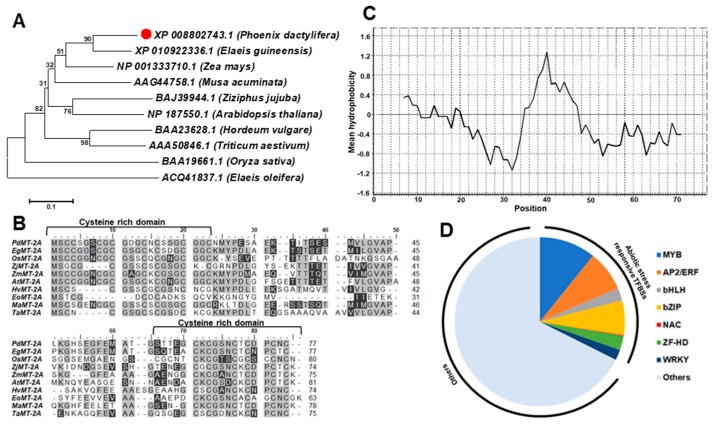
Sequence analysis of 10 metallothioneins (MT) amino acid sequences of various plant species and the phylogenetic tree constructed using the neighbor-joining method. The bootstrap values on the nodes represent percentages of 1000 repetitions (**A**). The multiple sequence alignment of the deduced amino acid sequence of PdMT2A and other MT2A isoforms from other plant species. The gray-colored highlighting represents identical and conserved regions and the dark highlighting represents similar regions with one amino acid difference. The two cysteine-rich domains are indicated at the N- and C-termini of the sequences (**B**). The hydrophobicity plot of each amino acid in the PdMT2A sequence according to the Kyte–Doolittle hydrophobicity scale (**C**). The sequence analysis of the putative 2000-bp promoter region upstream of the *PdMT2A* start codon, showing the abundance of abiotic-stress-related transcription factor binding sites (TFBSs) within the putative promoter sequence (**D**).

Computational analysis revealed that *PdMT2A* (GenBank accession XM_008804521.2) is a 234-bp sequence, coding for a 77-amino-acid-long polypeptide with a molecular weight of 7.62 kDa and a theoretical isoelectric point (pI) of 4.38. The phylogenetic analysis indicated that the *Phoenix dactylifera* L. metallothionein 2A is closely related to that of *Elaeis guineensis* (African oil palm), followed by *Zea mays* L. (maize) and *Musa acuminate* (banana), which are clustered within the same clade (Figure 1A). The *PdMT2A* protein sequence analysis showed the presence of 14 cysteine residues which are located within the cysteine-rich domain, one each at the N- and C-termini, which is consistent with metallothionein proteins from other plant species (Figure 1B). The mean hydrophobicity plot, according to the Kyte–Doolittle scale, showed that a large portion of the protein is hydrophilic (Figure 1C), which is also consistent with other previously characterized metallothioneins of the same class from other plant species. In order to predict the expression behavior of *PdMT2A* under different environmental conditions, the putative promoter sequence upstream of the start codon was computationally analyzed. The analysis revealed that the putative promoter consisted of 32% abiotic-stress-responsive transcription factor binding sites (TFBSs), amongst the total cis-regulatory elements analyzed in the sequence. The majority of TFBSs include myeloblastosis viral oncogene (MYB), apetala2/ethylene response factor (AP2/ERF) and basic domain-leucine zipper (bZIP) cis-regulatory elements, amongst which most of the AP2/ERF and MYB regulatory elements were located close to the start codon (Figure 1D). 

### 2.2. Reactivity of PdMT2A Protein Against DTNB In Vitro

**Figure 2:**
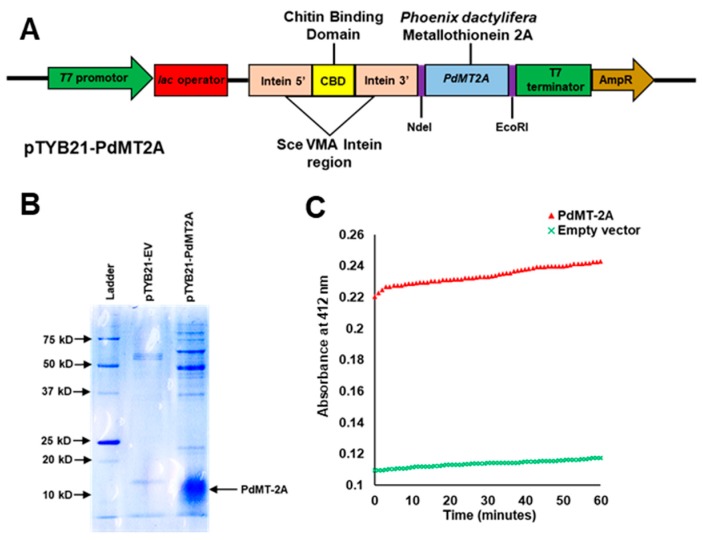
Production of recombinant PdMT2A protein in *E. coli.* Schematic representation of the expression vector (**A**). The polyacrylamide gel image of the partially purified recombinant PdMT2A protein and the protein produced by the pTYB21 empty vector in *E. coli*. (**B**). The reactivity of the recombinant PdMT2A protein and the empty vector against the 5,5′-dithiobis-2-nitrobenzoic acid (DTNB) substrate shows the initial speed of the reaction from the plot of absorbance versus time (**C**).

In order to determine the molecular function of PdMT2A, the *PdMT2A* gene was cloned into pTYB21 plasmid (Figure 2A), overexpressed in *Escherichia coli* and purified using affinity chromatography. However, due to the small size and the instability of the protein, partially purified recombinant PdMT2A protein was obtained. The reactivity of the PdMT2A recombinant protein with 5,5′-dithiobis-2-nitrobenzoic acid (DTNB) was tested, against the proteins from cells harboring a pTYB21 empty vector as a negative control (Figure 2B). This assay showed that the PdMT2A protein exhibited a reactivity about twice as high as the negative control. The time course of affinity showed that the PdMT2A protein had a high initial reaction speed for the reaction with DTNB (Figure 2C).

### 2.3. PdMT2A Confers Drought and Oxidative Stress Tolerance in Yeast

**Figure 3:**
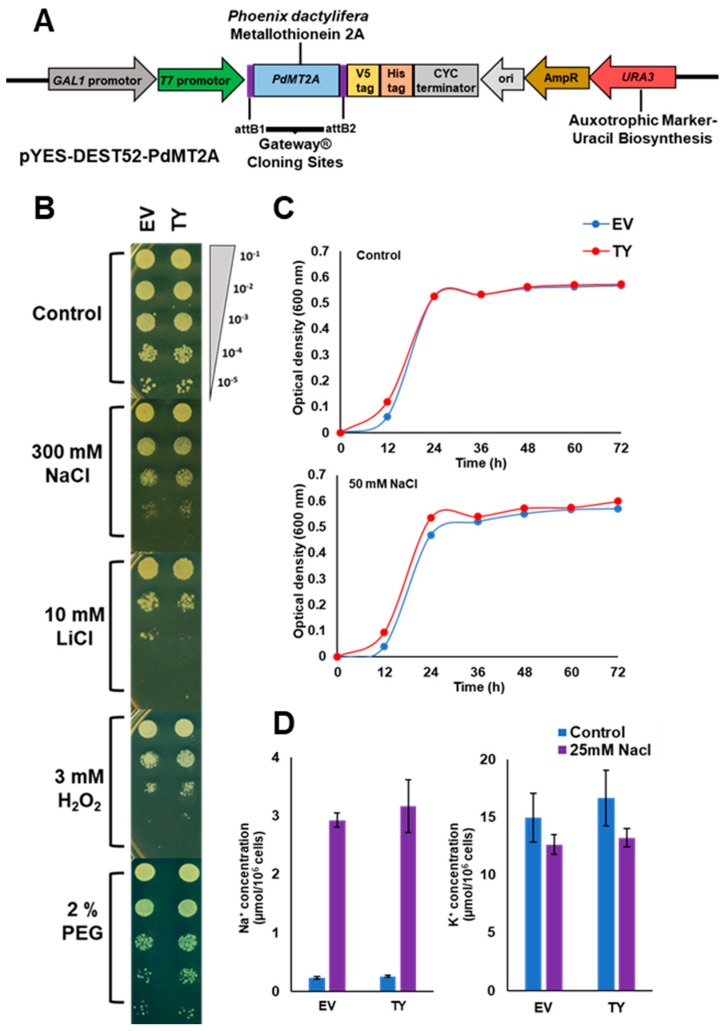
Overexpression of *PdMT2A* in yeast. Schematic representation of the cloned *PdMT2A* gene within the pYES-DEST52 plasmid (**A**). The effect of the *PdMT2A* transgene on the growth of yeast cells and the relative tolerance of transgenic (TY) and empty vector (EV) cells tested by yeast spot assay when grown under control and various abiotic stress conditions on solid media (**B**). Yeast liquid culture assay used to test the relative tolerance of TY and EV cells grown under control and 50 mM NaCl stress conditions (**C**). Accumulation of Na^+^ and K^+^ in TY or EV cells when grown under control and 25 mM NaCl salinity stress conditions (**D**). The bars represent the mean concentration of Na^+^ and K^+^ (± SE, *n* = 3).

The *PdMT2A* gene was cloned into yeast expression vector pYES-DEST52 (Figure 3A) and transformed into yeast cells, in order to test its function in a basic eukaryotic system. Initially, the recombinant (TY) and the empty plasmids (EV) were transformed into the wild-type yeast strain INVSc1 as a control in the yeast assays. The comparative spot growth responses between TY and EV under different abiotic stresses were assayed, but no differences were observed (Figure S1). As a result, we decided to use the salt-sensitive yeast strain BYT458. The yeast spot assay showed that both transgenic (TY) and empty vector (EV) cells had uniform growth under control conditions. However, the TY cells had enhanced growth on plates containing PEG (polyethylene glycol) and H_2_O_2_, suggesting the involvement of *PdMT2A* in drought and oxidative stress tolerance. On plates supplemented with LiCl, the EV cells showed slightly better growth than the TY cells, whereas on the plates containing 300 mM NaCl, the TY and EV cells did not show visible differences in growth pattern (Figure 3B). The pattern of yeast growth was also tested in liquid synthetic medium (LSM) supplemented with 50 mM NaCl. NaCl concentrations of 300 mM and 50 mM were selected for these assays because they were the highest sustainable concentrations for this strain of yeast when grown on solid and liquid media, respectively. The TY cells showed a high growth rate during the lag and exponential growth phases, compared to the EV cells (Figure 3C). Concentrations of LiCl and H_2_O_2_ as small as 1 mM and 0.5 mM, respectively, were detrimental for yeast in liquid culture. However, sorbitol concentration as high as 1 M did not lead to differences in the growth patterns, and therefore further liquid culture assays were not performed for these stresses.

The effect of *PdMT2A* on the Na^+^ and K^+^ balance was tested by measuring Na^+^ and K^+^ concentrations in the TY and EV cells grown in LSM supplemented with 25 mM NaCl. Although Na^+^ and K^+^ concentrations were elevated under salt stress, no significant differences were observed between TY and EV cells (Figure 3D).

### 2.4. Arabidopsis Seedlings Overexpressing PdMT2A Exhibit Improved Drought and Salinity Tolerance

**Figure 4:**
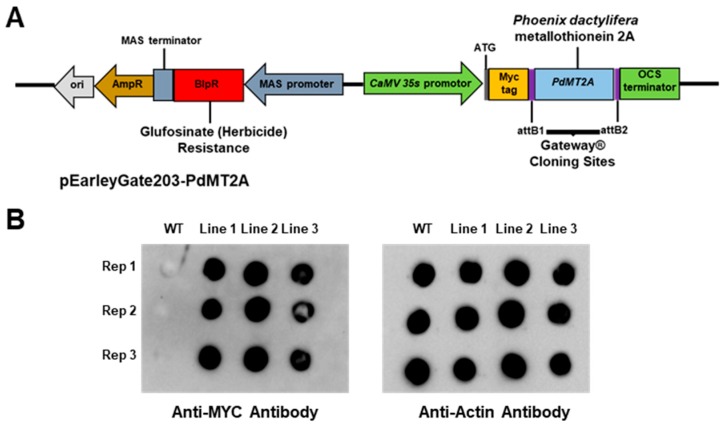
Overexpression of *PdMT2A* in *Arabidopsis*. Schematic representation of the cloned *PdMT2A* gene within the pEarleyGate 203 plasmid (**A**). Dot-blot immunoassay using total protein lysate extracted from the transgenic and the wild-type (WT) *Arabidopsis* plants, to demonstrate the heterologous expression of Myc-PdMT2A protein and the actin protein, using anti-Myc and anti-actin antibodies, respectively (**B**).

The *PdMT2A* gene was cloned downstream of the Myc epitope tag of the binary vector (pEarleyGate 203) and transformed into *Arabidopsis* using the *Agrobacterium*-mediated transformation method (Figure 4A). The presence of the transgene in the transgenic lines was verified using PCR. In addition, a dot-blot immunoassay was performed to detect the accumulation of the heterologous PdMT2A protein in the transgenic plants, using anti-Myc antibodies. The immunoassay results detected a strong signal in the transgenic *Arabidopsis* lines but none in the wild-type (WT) *Arabidopsis* (Figure 4B). A strong signal was detected for the actin protein in WT and transgenic lines using anti-actin antibodies, which served as a loading control (Figure 4B). These results confirmed the expression of PdMT2A protein in transgenic plants.

**Figure 5:**
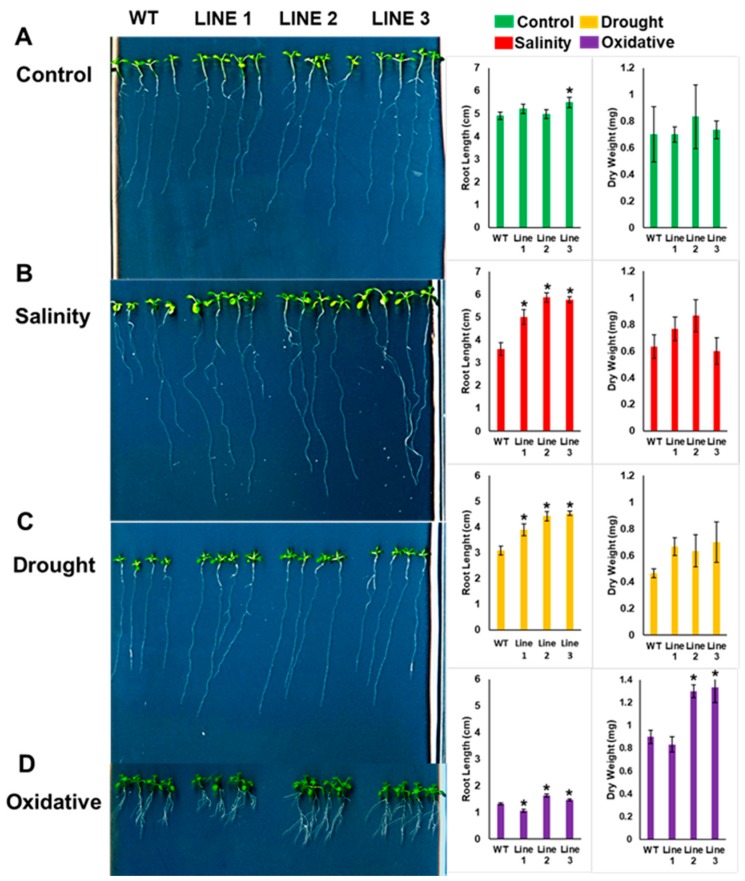
The effect of *PdMT2A* on the phenotype of the transgenic *Arabidopsis* seedlings. The growth pattern, root length and dry weight of the WT and *PdMT2A* transgenic *Arabidopsis* lines were measured when the seedlings were grown on plain half-MS plates as a control (**A**) or on half-MS plates supplied with 100 mM NaCl (**B**), 150 mM mannitol (**C**) or 2 mM H_2_O_2_ (**D**), for 14 days. The length of the main root was measured manually using a standard centimeter scale. The bars represent either the mean root length in cm or the dry weight in mg (± SE, *n* = 3), while the asterisks indicate a significant difference from WT plants (*p* < 0.05).

The abiotic stress tolerance of the transgenic *Arabidopsis* seedlings was assayed on plain half-strength Murashige and Skoog (MS) agar medium containing plates, or on MS agar plates supplemented with NaCl, mannitol or H_2_O_2_, representing salinity, drought and oxidative stresses, respectively. The three independent transgenic lines grown on NaCl, mannitol and H_2_O_2_ plates had significantly (*p* < 0.05) longer roots, larger-sized leaves and greater biomass accumulation, compared to the respective WT plants (Figure 5). Unlike other stress conditions, where only the elongation of the main root was observed, a unique pattern of short main roots coupled with heavy branching of the root system was observed under H_2_O_2_ stress (Figure 5D). The dry mass accumulation pattern was relatively greater in transgenic *Arabidopsis* lines subjected to drought and H_2_O_2_ treatment, compared to those under salt stress. This observation is consistent with the yeast analysis, where the transgenic yeast also displayed a better tolerance under drought and oxidative stresses compared to salt stress.

**Figure 6:**
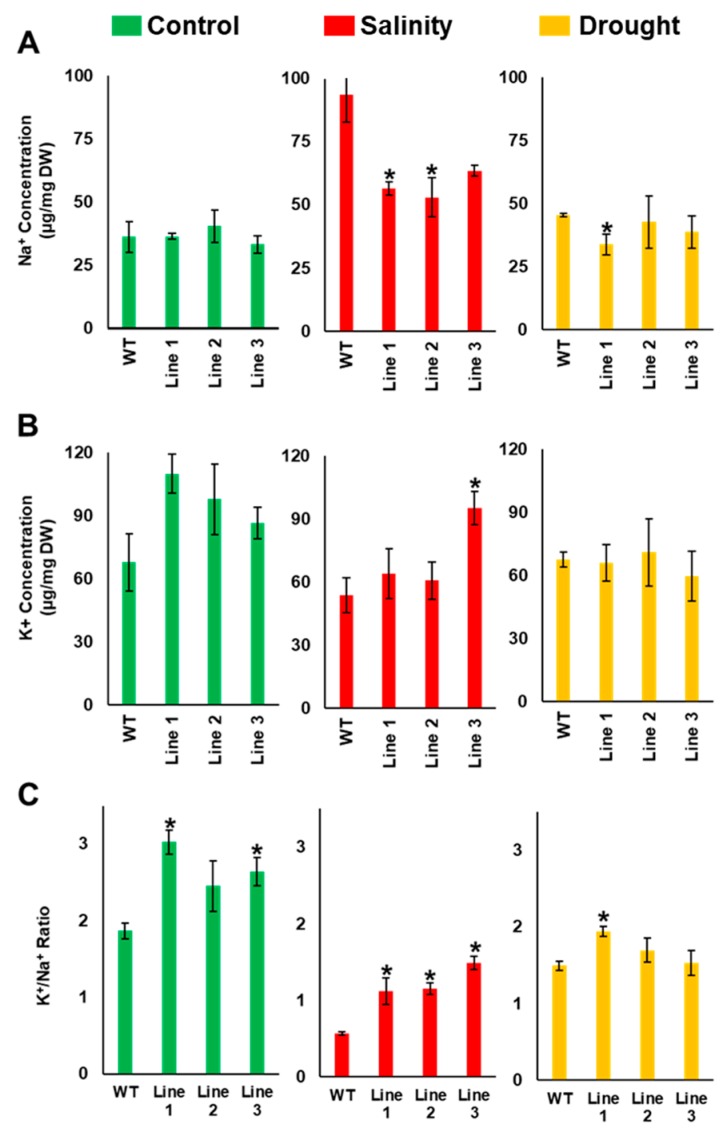
The effect of *PdMT2A* on the accumulation of Na^+^ and K^+^ content in *Arabidopsis*. Accumulation of Na^+^ (**A**) and K^+^ (**B**) and the K^+^/Na^+^ ratio (**C**) in *PdMT2A* transgenic and WT *Arabidopsis* plants when subjected to control, drought (150 mM mannitol) and salinity (100 mM NaCl) conditions on half-strength MS-medium plates. The bars represent the mean concentrations of Na^+^ and K^+^ (± SE, *n* = 3), while the asterisks indicate a significant difference from the WT plants (*p* < 0.05).

The Na^+^ and K^+^ concentrations in the stressed and non-stressed transgenic and wild-type seedlings were measured to determine the effect of the transgene on Na^+^ and K^+^ homeostasis during abiotic stress conditions. As expected, Na^+^ concentration did not vary between transgenic and WT seedlings under control and drought conditions. On the other hand, the Na^+^ concentration of transgenic lines was significantly (*p* < 0.05) lower than that of the WT plants under salinity (Figure 6A). Interestingly, the transgenic lines had a higher K^+^ concentration than the WT plants under control conditions (Figure 6B). Furthermore, under both salinity and drought conditions, the transgenic lines tended to accumulate more K^+^ during salt stress than the WT lines (Figure 6B). This was further evident from the K^+^/Na^+^ ratio analysis. The K^+^/Na^+^ ratio in three independent transgenic lines was significantly (*p* < 0.05) higher than in the corresponding WT plants under salinity. Albeit at slightly lower levels, a similar trend of higher K^+^/Na^+^ ratio was also observed under both control and drought stress conditions in transgenic lines, compared to WT plants (Figure 6C).

**Figure 7:**
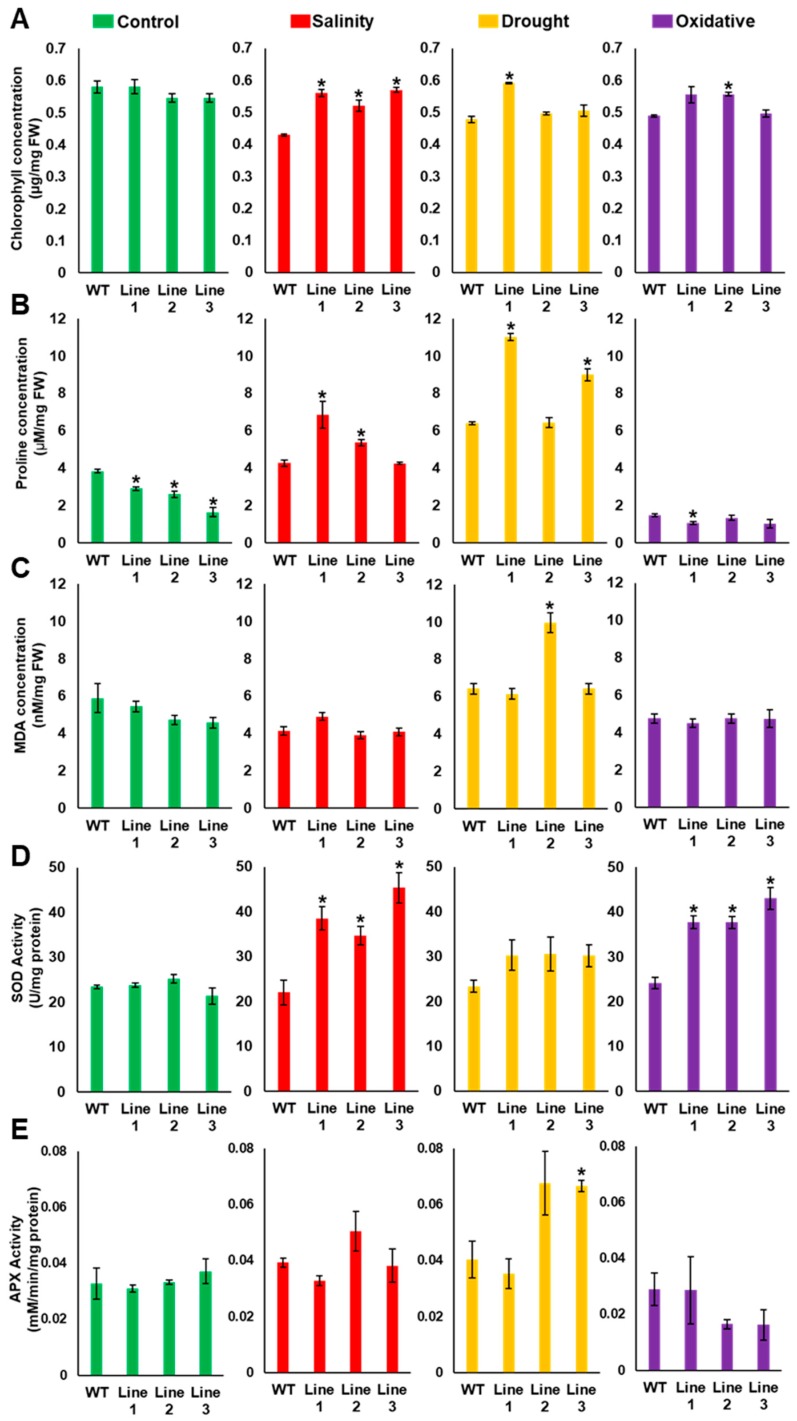
The effect of *PdMT2A* on chlorophyll, proline and antioxidant enzymes. The effect of drought, salinity and oxidative stress on the chlorophyll content (**A**), proline concentration (**B**), malondialdehyde (MDA) concentration (**C**), superoxide dismutase (SOD) activity (**D**) and ascorbate peroxidase (APX) activity (**E**) of *PdMT2A* transgenic and WT *Arabidopsis* plants. The bars represent the mean chlorophyll, proline or MDA concentration and the SOD or APX activity (± SE, *n* = 3), while the asterisks indicate a significant difference from the WT plants (*p* < 0.05).

The effect of abiotic stress on photosynthesis was determined by measuring the chlorophyll levels. The transgenic lines had significantly (*p* < 0.05) higher levels of chlorophyll under salinity, compared to the WT plants. Moreover, under drought and oxidative stress conditions, chlorophyll retention was greater in transgenic lines (Figure 7A). 

The proline accumulation in transgenic lines under drought and salinity was high, although only two transgenic lines showed significantly (*p* < 0.05) higher accumulation than the WT plants. Conversely, the proline concentration was low in all transgenic lines under oxidative stress, compared to the WT plants (Figure 7B). Interestingly, under control conditions, the proline concentration was significantly lower in the transgenic plants compared to the WT plants.

Lipid peroxidation levels and superoxide dismutase (SOD) and ascorbate peroxidase (APX) activities were analyzed to determine the effect of the transgene on the status of the antioxidant machinery under abiotic stress conditions. To assess the effect of stress on the cell membrane status, lipid peroxidation was estimated by measuring the amount of malondialdehyde (MDA) in the samples. The MDA levels were higher in only one of the transgenic lines subjected to drought stress (Figure 7C). The SOD activity was relatively high in all transgenic lines under salinity, drought and oxidative stress conditions, although the activity was significantly (*p* < 0.05) higher under both salinity and oxidative stress but not under drought (Figure 7D). The APX activity of the transgenic and WT plants was similar under control conditions. However, under drought, the transgenic *Arabidopsis* had significantly (*p* < 0.05) higher APX activity than WT plants. The APX enzyme activity under salinity and oxidative stress was inconsistent among the three transgenic lines and the WT plants (Figure 7E).

### 2.5. The Soil-Grown Transgenic Arabidopsis Lines Displayed Improved Stress Tolerance 

**Figure 8:**
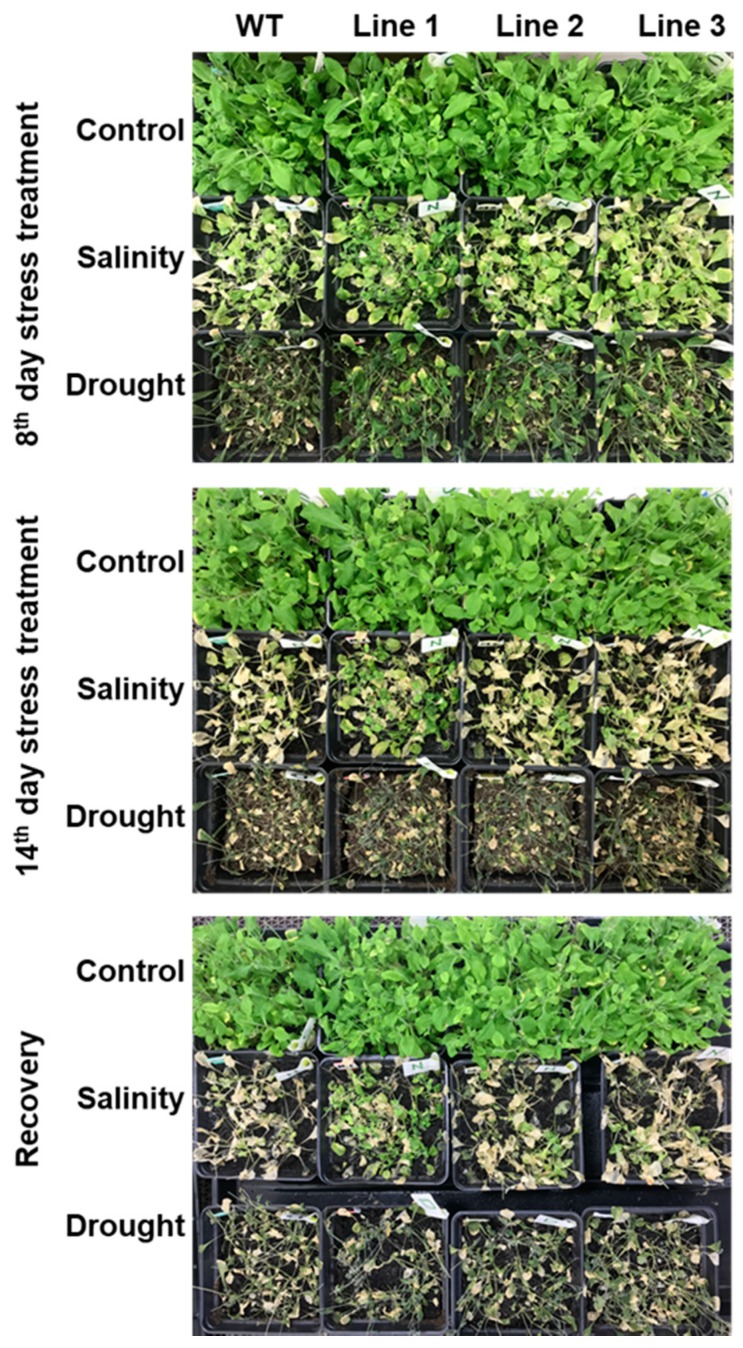
Performance of the *PdMT2A* transgenic and WT *Arabidopsis* plants grown on soil and subjected to salinity (200 mM NaCl) and drought stress for 14 days.

The transgenic lines and the WT *Arabidopsis* plants were grown on soil to assess their response to drought and salinity. Both transgenic and WT plants showed healthy growth under control conditions; however, the three transgenic lines showed an improved tolerance to drought and salinity by the eighth day of the stress treatment, compared to WT plants. The transgenic lines continued to perform better than the WT plants under salt stress even on the 14th day of the salt-stress treatment. On the other hand, by the 14th day of drought stress, both the transgenic and WT plants were completely wilted, suggesting that the stress intensity was severe. In order to determine the recovery capacity of the transgenic and WT plants after 14 days of stress treatment, both drought- and salinity-stressed plants were allowed to recover from their respective stress treatments. A small fraction of salt-stress-treated transgenic lines were able to recover but not the WT plants. Neither the transgenic lines nor the WT plants were able to recover from the drought treatment (Figure 8).

**Figure 9:**
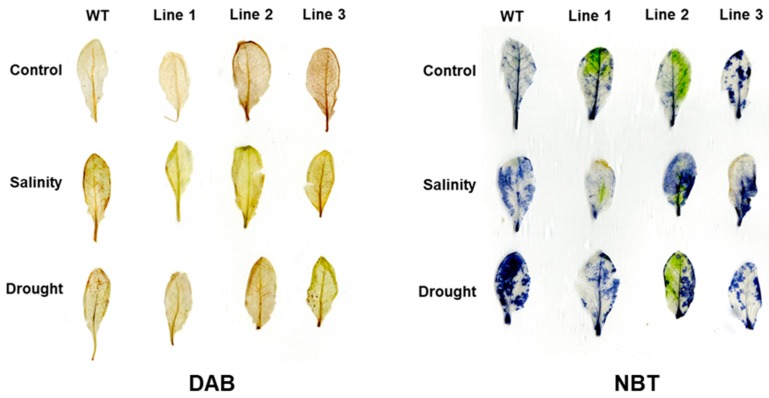
The 3,3-diaminobenzidine (DAB) and nitro blue tetrazolium (NBT) histochemical staining of WT and transgenic *Arabidopsis* plant leaves under salinity and drought stress conditions.

The accumulation of H_2_O_2_ and superoxide anion (O_2_^−^) was detected using 3,3-diaminobenzidine (DAB) and nitro blue tetrazolium (NBT) staining. The leaves of WT plants displayed darker spots than the transgenic *Arabidopsis* lines under salinity and drought stress conditions, which clearly indicates high accumulation of H_2_O_2_ and O_2_^−^ in WT plants. The transgenic lines exhibited slightly lower accumulation of H_2_O_2_ under drought and salinity as indicated by the DAB staining. Additionally, the NBT staining clearly indicates a lower accumulation of O_2_^-^ in transgenic plants, compared to the WT plants. This indicates that the transgenic plants had a lower accumulation of ROS, compared to the WT plants (Figure 9).

The qPCR analysis was performed to determine the effect of the *PdMT2A* transgene on the expression level of some other genes involved in salinity tolerance. The expression level of genes associated with salt tolerance (e.g., Na^+^ uptake/accumulation/extrusion), was assessed in the transgenic lines compared with WT plants under stress conditions. We determined the expression level of *CHX20*, *SOS1*, *HKT1*, and vacuolar Na^+^/H^+^ antiporter as well as *ABA* and *SOD* genes as they are known to be involved in salinity stress tolerance mechanisms in plants.

**Figure 10:**
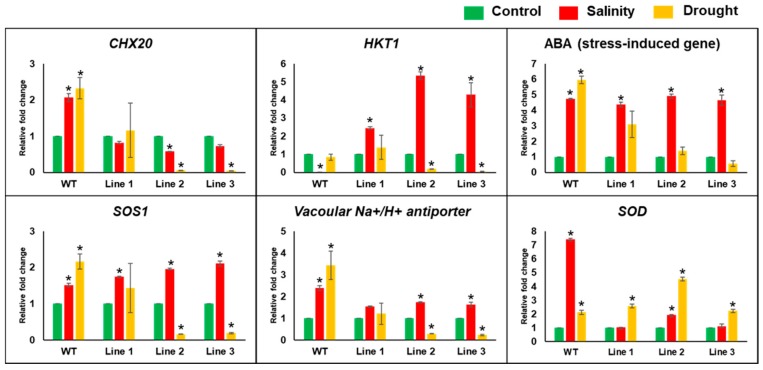
Gene expression analysis of CHX20, SOS1, HKT1, vacuolar Na^+^/H^+^ antiporter, ABA stress-induced gene and SOD in WT and transgenic *Arabidopsis* subjected to salinity and drought stress. The bars represent the relative fold change (± SE, *n* = 3), while the asterisks indicate a significant difference from the control (*p* < 0.05).

The results showed that the expression of a putative cation/H+ exchanger (*CHX20*) and a vacuolar Na^+^/H^+^ antiporter gene was downregulated in transgenic lines, compared to the WT plants under drought and salinity. Similarly, the expression of salt overly sensitive 1 gene (*SOS1*) and ABA stress-induced gene (*ABA*) was downregulated under drought stress. However, the high-affinity K^+^ transporter 1 (*HKT1*) and *SOD* genes were upregulated in transgenic lines compared to WT plants, under salinity stress (Figure 10).

## 3. Discussion

Date palm is a relatively stress-tolerant plant, but it has been lately observed that drought and salinity affect the health of the plant to a remarkable extent, which reduces the productivity. To obtain molecular insights into the salinity tolerance mechanisms of date palm, various approaches, including transcriptome, methylome, metabolome and microbiome global analyses, have recently been employed [7,11,12,14,40,41]. Functional yeast analysis of the cDNA library was undertaken, which resulted in the identification of several genes (including the date palm *PdMT2A* gene) that could play a critical role in salinity tolerance in date palm [18]. MTs are well known for detoxification of heavy metal homeostasis [28,42,43]. However, MTs have also been shown to play a role in drought and salinity tolerance [33,44]. In this study, an attempt was made to functionally characterize the importance of the *PdMT2A* gene in salt, drought and oxidative stress tolerance.

The deduced amino acid sequence of the *PdMT2A* gene shares common features with other metallothioneins, previously characterized from other plant species. These features include the presence of specific cysteine residues and the hydrophobicity profile. In addition, the phylogenetic analysis showed a high similarity between the *PdMT2A* gene and its orthologs in other plant species, and therefore members of this gene family in the plants share a common ancestor gene (Figure 1A). These results suggest that MTs form part of the basic stress tolerance mechanisms in plants [29,33]. In fact, this evolutionary relationship amongst the plant MTs was demonstrated earlier in the genome-wide study of MT-family genes from *Arabidopsis thaliana* and various *Brassica* species [28]. Consistently, the putative promoter region of *PdMT2A* has been found to possess a significant percentage of abiotic-stress-responsive TFBSs [45]. Amongst the TFBSs, MYB, bZIP, AP2/ERF and WRKY are known to be involved in abiotic stress responses in plants [46,47,48,49,50] (Figure 1D). Previously, transcriptome analysis of the leaf and root tissues of date palm showed that various genes of MYB, AP2/ERF, WRKY, NAC and bHLH transcription-factor families were co-overexpressed in response to salinity, and the abundance of these TFBSs was higher in root tissues than in the leaf tissues [12]. Interestingly, the *TaMYB2* gene of wheat is used as a molecular marker for drought stress tolerance in common wheat [51]. The characterization of the *HaAP2/ERF* transcription-factor family showed that the *AP2/ERF*-family genes were overexpressed as a result of drought, cold, salt and heat treatments [52]. Additionally, the watermelon *ClWRKYs* had a diverse response to abiotic stresses, which suggests that WRKYs positively or negatively participate in plant tolerance of drought, salt or cold stress [53]. These observations argue in favor of the potential involvement of *PdMT2A* in abiotic stresses and are consistent with previous observations on plant MTs [34,54,55].

The recombinant PdMT2A protein produced in *E. coli* had high initial velocity of the reaction when DTNB was used as a substrate. The MTs are one of the main sources of thiol in cells, and members of the thiol group are preferred targets for free radicals, which can be compared to the reduction of the disulfide bond of DTNB [56]. In addition, the cysteine residues in the proteins, like MTs, are highly reactive towards oxidizing agents including ROS [57]. Hence, the high initial speed of the reaction might be correlated with the high antioxidant capability of the PdMT2A protein (Figure 2C). However, a detailed study regarding the chemistry and protein folding of PdMT2A is required.

When *PdMT2A* was overexpressed in yeast, it did not enhance the growth on solid media supplemented with NaCl, suggesting that *PdMT2A* is not part of the salt tolerance mechanism in yeast (Figure 3B). A similar pattern for the growth rate of yeast cells was observed previously when the *rgMT* gene of rice was overexpressed in yeast and assessed for salt tolerance [58]. Unlike in solid media, when the yeast cells were grown in liquid media (LSM) supplemented with NaCl, the TY cells showed slightly enhanced growth rates in the lag and the exponential growth phase compared to EV cells (Figure 3C). This enhanced growth rate could possibly be due to elevated early detoxification of the ROS in the TY cells in the liquid medium [34]. Apparently, there was no significant difference in the accumulation of Na^+^ and K^+^ by yeast cells under salinity stress. These results suggest that PdMT2A might not play a significant role in maintaining the Na^+^ and K^+^ balance in yeast cells (Figure 3D). This finding is consistent with a previous study done on yeast cells transformed with the *SsMT2* gene of *Suaeda salsa* L. [24].

The TY yeast cells displayed enhanced growth rates under oxidative and drought stresses (Figure 3B), suggesting that the transgenic yeast overexpressing *PdMT2A* possesses enhanced antioxidant activity. These observations were consistent with previous reports in which overexpression of the *GhMT3a* gene from cotton led to an enhanced tolerance of H_2_O_2_ toxicity [34], and the overexpression of the *SsMT2* gene from *Suaeda salsa* L. led to an enhanced tolerance of salinity and oxidative stress in yeast [24]. In addition, in transgenic *E. coli* cells, the *OsMT3a* gene of rice confers salinity and heavy metal stress tolerance [29].

The transgenic *Arabidopsis* seedlings had longer roots and slightly larger leaves, coupled with a higher biomass accumulation, compared to WT plants under drought, salinity and oxidative stress conditions (Figure 4). These observations support the notion that *PdMT2A* plays a role in abiotic stress tolerance, as previously reported for other plant MTs [24,33].

Plants maintain a stable regulation of Na^+^ and K^+^ homeostasis in the cytosol in the normal functioning of cellular and physiological processes [59]. The Na^+^ accumulation in *PdMT2A* transgenic and WT plants was similar under control conditions; however, under salinity the transgenic plants had a significantly (*p* < 0.05) lower Na^+^ concentration and a higher K^+^/Na^+^ ratio than WT plants. These results are similar to those of an earlier report in which *SsMT2* overexpression was shown to have similar effects [24]. Plants have various strategies to modulate the influx and efflux of Na^+^ and K^+^, including ion transporters such as NHX, CHX, HKT and Na^+^/H^+^ antiporters [41]. The plant MTs including *PdMT2A* are not previously known to play a role in Na^+^ and K^+^ transportation, however differential accumulation of Na^+^ and K^+^ ions was observed as a result of the overexpression of *PdMT2A.* Therefore, we ask what could contribute to the low Na^+^ accumulation in transgenic lines, compared to WT plants grown under salt stress. We speculate that the abundant *PdMT2A* could indirectly decrease the Na^+^ uptake, increase the activity of transporters that pump Na^+^ out from the plant cells or affect both processes. Indeed, gene expression analysis revealed the regulatory effect of *PdMT2A* on some ion transporter genes (Figure 10) under stress. This effect may modulate the accumulation of Na^+^ and K^+^ ions in transgenic plants. For example, the downregulation of *CHX20* and upregulation of *SOS1* in transgenic plants under salinity could be a reason for the reduction of Na^+^ ions concentration. However, upregulation of *HKT1* under salinity in the transgenic plants might have contributed to the accumulation of K^+^ ions. Hence, this differential gene expression might be responsible for the balanced K^+^/Na^+^ ratio found in the transgenic plants under salinity. This notion may suggest a role for the PdMT2A in gene expression regulation probably through the activation of transcription factors such as the previously reported zinc finger transcription factor, which was regulated by metallothionein–thionein conjugate pair [39]. However, further studies are needed to determine the exact reason for lower Na^+^ accumulation in the transgenic lines overexpressing *PdMT2A*.

The higher total chlorophyll content of the transgenic plants compared with the WT plants under abiotic stresses, especially under salinity stress, implies that the integrity of the photosynthesis system was maintained in the transgenic lines, due to the *PdMT2A* transgene (Figure 7A). A previous study also reported an increase in chlorophyll content under drought, salinity and heavy metal stress, in transgenic tobacco overexpressing the *SbMT2* gene [60]. In addition, the *TaMT3* gene of *Tamarix androssowii* led to high chlorophyll content in transgenic tobacco under heavy metal stress [43].

Proline is an osmoprotectant that maintains turgor pressure inside the plant cells during dehydration and high salinity stresses and also protects plants from oxidative damage. Hence, an increase in proline content under stress is considered to be an important phenomenon [61,62]. In this study, the proline concentration in the transgenic *Arabidopsis* lines was significantly (*p* < 0.05) high under drought and salinity (Figure 7B). Intriguingly, proline concentration was significantly (*p* < 0.05) lower in the transgenic lines grown under control conditions. Previous studies have reported the antioxidant ROS-scavenging activity of proline [63]. Therefore, the presence of a heterologous ROS scavenger such as PdMT2A in transgenic plants may have led to the reduction of proline under non-stress conditions. The histochemical staining for ROS detection also showed a reduction in ROS accumulation in transgenic plants under drought and salinity (Figure 9). This may imply an important role of *PdMT2A* in reducing the ROS routinely produced in the plants due to various biochemical reactions. This may also affect the energy conservation strategy of plants by manipulating their metabolism.

Under stress conditions, the fact that plants tend to accumulate ROS and maintain ROS homeostasis via scavenging enzymes such as SOD and APX, is well documented [64]. The overexpression of *PdMT2A* in *Arabidopsis* increased the SOD activity of transgenic plants compared to WT plants, under drought, salinity and oxidative stresses. This was consistent with previous reports in which high SOD expression was observed under NaCl or osmotic stress conditions in transgenic tobacco overexpressing the *SbMT2* gene [60]. The greater SOD levels probably reflect greater ROS-scavenging activity, imparting improved abiotic stress tolerance (Figure 7D). The APX activity in transgenic Arabidopsis did not vary compared with WT plants, under salinity and oxidative stress conditions. However, there was an increasing trend of the APX activity in transgenic plants subjected to drought stress (Figure 7E), and this trend was also observed in transgenic tobacco expressing *SbMT2* [60]. 

## 4. Materials and Methods

### 4.1. Sequence Analysis of Date Palm Metallothionein

*PdMT2A* homologous sequences from other plant species were retrieved from the NCBI database (https://www.ncbi.nlm.nih.gov/) for the sequence comparison. The sequences were aligned using ClustalW and a phylogenetic tree was constructed using the neighbor joining method implemented via the MEGA X software package [65]. The physicochemical properties of the PdMT2A protein were analyzed using the ProtParam tool (https://web.expasy.org/protparam/) and the mean hydrophobicity index was plotted according to the Kyte–Doolittle scale [66,67]. The putative 2000-bp upstream promoter sequence was analyzed for cis-binding sites using PlantPAN 2.0 (http://PlantPAN2.itps.ncku.edu.tw/) [68]. The results were analyzed, and a pie diagram representing the distribution of transcription factor binding sites (TFBSs) was produced.

### 4.2. Recombinant Protein Production and Reaction with DTNB

The *PdMT2A* transcript was amplified via PCR from the date palm cDNA using forward (5′-GGATTTCCATATGATGTCTTGCTGTAGCGG-3′) and reverse (5′-GGATTTCCATATGATGTCTTGCTGTAGCGG-3′) primers. The amplicon was designed to include the Nde1 and EcoR1 restriction sites, in order to be compatible with the same sites in the pTYB21 plasmid (New England Biolabs, Ipswich, MA, USA). The resulting plasmid construct was named pTYB21-PdMT2A. After amplification in the *Escherichia coli* DH10B strain, the construct was transformed into the *E. coli* ER2566 strain using the standard electroporation method, to produce the recombinant protein. A selected transformed *E. coli* colony was inoculated into 0.5 L of Luria-Bertani (LB) medium for 16 h at 37 °C with agitation at 200 rpm. Recombinant protein production was induced by adding 250 µL of 1 M isopropyl β-d-1-thiogalactopyranoside (IPTG) and incubating at room temperature for 16 h with agitation at 200 rpm. Subsequently, the recombinant protein purification was carried out via in-column affinity chromatography with chitin beads, using an Impact™ Kit and following the manufacturer’s instructions (New England Biolabs). In addition, 1 mM ethylenediaminetetraacetic acid (EDTA) was included in the protein extraction buffer in order to reduce the metal binding prior to the reaction with the substrate. The intein tag contains a chitin-binding domain (CBD) for affinity purification of the fusion protein on a chitin resin and was cleaved using dithiothreitol (DTT), which also prevents the formation of disulfide bridges between groups of cysteine residues of the PdMT2A. The amount of protein recovered was quantified using the standard curve of glutathione (GSH), as described earlier [69]. The reactivities of the recombinant PdMT2A protein and the corresponding empty vector control protein, were measured in a 100-µL reaction vessel containing 0.5 mM 5,5′-dithiobis-2-nitrobenzoic acid (DTNB) as a substrate, 0.2 M phosphate buffer (pH 8.0) and 0.5 mM of the recombinant protein [70]. The formation of 2-nitro-5-thiobenzoic acid (TNB) was detected by monitoring the absorbance at 412 nm for 60 min. The initial speed of the reaction was obtained by plotting a curve of absorbance against time, using a spectrophotometer, as previously described [71].

### 4.3. Expression of PdMT2A in Salt-Sensitive Yeast Strain

The full-length *PdMT2A* gene was obtained from the date palm cDNA library and cloned into the yeast expression vector pYES-DEST52 (Thermo Fisher Scientific, Carlsbad, CA, USA), using site-specific Gateway™ recombination technology (Thermo Fisher Scientific) downstream of the galactose-inducible *GAL1* promoter (Figure 3A). The recombinant pYES-DEST52-PdMT2A vector (TY) and the empty pYES-DEST52 vector (EV) were transferred to the salt-sensitive mutant yeast (*S. cerevisiae*) strain BYT458 [72] (kindly provided by Hana Sychrova, Czech Republic), using Yeastmaker™ Yeast Transformation System 2 (Clontech Laboratories, Inc., Mountainview, CA, USA), following the manufacturer’s instructions. The yeast cells were grown in liquid synthetic medium (LSM) supplemented with 2% glucose. Subsequently, the yeast cells were spotted on solid synthetic medium (SSM) supplemented with 2% galactose, with 2% PEG (polyethylene glycol), 300 mM NaCl (sodium chloride), 10 mM LiCl (lithium chloride) or 3 mM H_2_O_2_ (hydrogen peroxide), as drought, salinity, Li toxicity and oxidative stresses, respectively. Ten-µL drops of serially diluted yeasts were spotted on the SSM plates and the plates were incubated at 30 °C for five days before the observations were made [18]. The growth rates of TY and EV cells were monitored by growing the cells in 20 mL of LSM supplemented with 2% galactose and 50 mM NaCl and incubated in a shaker-incubator at 30 °C with agitation at 200 rpm. The optical density (OD) of the liquid was measured every 12 h for three days. Similarly, to monitor the uptake and accumulation of Na^+^ and K^+^ in yeast, the TY and EV cells were grown in LSM supplemented with 25 mM NaCl, and Na^+^ and K^+^ concentrations were measured using a flame photometer, as described previously [73].

### 4.4. Generation of Transgenic Arabidopsis Plant Overexpressing PdMT2A Gene

A full-length *PdMT2A* gene was cloned in the binary plant expression vector pEarleyGate 203 (TAIR stock ID—CD3-689) in a frame with an Myc-tag protein and overexpressed under the control of the *35S CaMV* constitutive in-plant promoter. The pEarleyGate 203-PdMT2A construct was amplified in the *E. coli* DH10B strain and further transformed into *Agrobacterium tumefaciens* LBA4404 strain by electroporation. To produce transgenic *Arabidopsis* lines, 45-day-old wild-type (WT) *Arabidopsis thaliana* Columbia (Col-0) plants were transformed using the standard Agrobacterium-mediated floral dip method [74]. The T0 seeds were harvested from the plants, vernalized at 4 °C and sown on soil. The transgenic plants were selected by spraying T1 plants with 0.01% Basta^®^ (Bayer, Germany) herbicide solution on the 6th and 10th day after gemination. The surviving transgenic plants (T1) were confirmed by PCR using *35S* promoter forward (5′-CAAGACCCTTCCTCTATATAAG-3′) and OSC terminator reverse (5′-CGCATATCTCATTAAAGCAG-3′) primers. The T2 seeds were harvested, dried, vernalized and germinated on half-strength Murashige and Skoog (MS) medium supplemented with 10 mg/L of Basta^®^ herbicide. The transgenic T2 lines, showing the 3:1 Mendelian segregation ratio for resistant:sensitive plants, were selected and planted in soil, and the T3 seeds were collected from them. Three independent homozygous transgenic lines were obtained by screening T3 seeds for 100% herbicide resistance and used for further experiments.

### 4.5. Detection of Overexpressed PdMT2A Protein in Arabidopsis Using Dot-Blot Immunoassay

The leaves of the transgenic *Arabidopsis* were harvested, flash-frozen and crushed to fine powder using liquid nitrogen. The total protein was extracted using 1 mL of extraction buffer composed of 100 mM tris-HCl (pH 7.5), 100 mM NaCl, 1 mM phenylmethyl sulfonyl fluoride (PMSF) and 5% glycerol. Since the PdMT2A is too small in size (approximately 8 kDa) and is unstable in air, it was not possible to detect this protein using the traditional Western blot assay. Therefore, the overexpressed PdMT2A protein fused to the Myc tag was detected via the dot-blot method using 0.2 µM PVDF membrane (Bio-Rad, Hercules, CA, USA), the anti-Myc-tag primary polyclonal antibody ab117499 (Abcam, Cambridge, UK) (dilution 1:1000) and the anti-mouse IgG (H&L) horseradish peroxidase (HRP) conjugated secondary antibody ab205719 (Abcam) (dilution 1:1000). As a protein loading control in the dot-blot assay, an immunoassay was carried out using the polyclonal anti-rabbit primary antibody AS13 2640 (Agrisera, Vännäs, Sweden) against the actin protein and the HRP-linked secondary anti-rabbit antibody AS09 602 (Agrisera). Immunoreactions were visualized by detecting the chemiluminescence signal on the immunoblot using Clarity enhanced chemiluminescence (ECL) substrate (Bio-Rad) and the image was visualized using the ChemiDoc™ Touch Imaging System (Bio-Rad).

### 4.6. Stress Treatment Assays of the Transgenic Arabidopsis Plants

The ability of the transgenic *Arabidopsis* lines to tolerate stress was compared with the wild-type (WT) *Arabidopsis* plants, both on plates containing MS medium (seedlings) and on soil (adult plants), in growth chambers maintained at 22 °C and 70% relative humidity (RH) with a 16-h-day/8-h-night cycle. For seedling assays, the seeds were initially germinated on plates with half-strength MS medium for four days, and later transferred to plates containing MS medium or plates supplemented with 100 mM NaCl, 150 mM mannitol or 2 mM H_2_O_2_, representing salinity, drought and oxidative stresses, respectively. Seedlings were grown for 14 days and then data (root lengths and dry weights) were collected. Similarly, for evaluating the stress tolerance of plants grown on soil, 21-day-old plants were either irrigated with 200 mM NaCl solution (salt stress) or watering was withheld for 14 days (drought stress).

### 4.7. Determination of Na^+^, K^+^, Chlorophyll, Proline and Malondialdehyde Content and Antioxidant Enzyme Activities

*Arabidopsis* seedlings were collected from the control, drought and salt-treated MS plates and dried, weighed and digested in 10 mL of a 0.1 M nitric acid solution before incubation in a shaker (100 rpm) for two days at room temperature. The mixture was filtered using Whatman No. 1 filter paper and the Na^+^ and K^+^ concentrations were measured using a flame photometer, as previously described [75]. The total chlorophyll content was measured using the 80% acetone-based method [76].

Proline concentration was determined by homogenizing plant tissue in 3% aqueous sulfosalicylic acid and allowing further reaction with acid ninhydrin and glacial acetic acid at 100 °C for one hour. The reaction mixture was cooled in ice and extracted by adding 1 mL of toluene followed by vortex mixing for 20 s. The absorbance was measured at 520 nm and the proline concentration was determined from a standard curve, as described previously [77]. Malondialdehyde (MDA), a product of lipid peroxidation, was determined spectrophotometrically, as described earlier [78]. Briefly, plant tissue was homogenized in 1 mL of 20% trichloroacetic acid containing 0.5% thiobarbituric acid in solution, and the mixture was heated to 95 °C for 30 min and centrifuged at 10,000 rpm for 10 min. The absorbance was measured at 532 nm and the value of the non-specific absorbance at 600 nm was subtracted. The concentration of MDA was calculated using its extinction coefficient of 155 mM^−1^·cm^−1^.

The crude enzyme extract was isolated from transgenic and WT *Arabidopsis* seedlings using an extraction buffer containing 50 mM potassium phosphate buffer (KPB) (pH 7.5), 1% polyvinylpolypyrrolidone (PVPP) and 2 mM EDTA. Superoxide dismutase (SOD) activity was determined based on inhibition of the photochemical reduction of nitro blue tetrazolium (NBT) with a reaction mixture containing 50 mM KPB, 75 mM NBT, 15 mM methionine, 2 mM riboflavin and 10 µL enzyme extract, when exposed to a 15-W fluorescent lamp for 10 min. The absorbance was recorded at 560 nm and the SOD activity was calculated as described earlier [79]. The ascorbate peroxidase (APX) enzymatic activity was determined by estimating the reduction of H_2_O_2_ by APX at 290 nm. The reaction mixture was composed of 50 mM KPB, 0.5 mM ascorbate, 0.1 mM H_2_O_2_ and 10 µL enzyme extract. The APX activity was determined using an extinction coefficient of 2.8 mM^−1^·cm^−1^ [80].

### 4.8. Detection of Superoxide Anion and H_2_O_2_ Accumulation in the Leaves of Arabidopsis

H_2_O_2_ accumulation was detected in leaves by staining with 3,3-diaminobenzidine (DAB), and superoxide anion (O_2_¯) was detected with nitro blue tetrazolium (NBT). Briefly, the stressed and non-stressed *Arabidopsis* leaves were immersed in 0.1% DAB solution and 0.2% NBT solution and incubated at room temperature overnight. The leaves were decolorized by immersing them in absolute ethanol and boiling for 15 min [81].

### 4.9. Expression Analysis of Abiotic-Stress-Responsive Genes using qPCR

RNA was extracted from the WT and transgenic *Arabidopsis* plants using a RNeasy Plant Mini Kit (Qiagen, Hilden, Germany), following the manufacturer’s instructions. A total of 100 ng of RNA was converted into cDNA using a SuperScript™ IV First-Strand Synthesis System (Invitrogen, Carlsbad, CA, USA), according to the manufacturer’s instructions. The quantitative real-time PCR (qPCR) gene expression analysis was performed for six stress-responsive genes, using gene-specific primer pairs (Table S1). A 20-fold diluted cDNA was used with SsoAdvanced™ Universal SYBR^®^ Green Supermix (Bio-Rad) for qPCR, in a CFX96 Touch™ Real-Time PCR Detection System (Bio-Rad). The *Arabidopsis* actin (AtActin accession number AT3G18780) was used as a reference gene to normalize the expression data, using the 2^−ΔΔ*C*t^ method [82].

### 4.10. Statistical Analysis

One-way analysis of variance (ANOVA) was used, with Tukey’s post hoc test, to determine the statistically significant differences (*p* < 0.05) between the means of tested parameters.

## 5. Conclusions

The study showed that the *PdMT2A* gene improved drought and salinity tolerance in yeast. In addition, the transgenic *Arabidopsis* plants performed better than the WT plants under drought, salinity and oxidative stress conditions. The transgenic plants had maintained a high K^+^/Na^+^ ratio which could be partly attributed to an indirect effect of the *PdMT2A* transgene on regulating the plant ion transporters. The transgenic plants maintained high chlorophyll retention under stress conditions, indicating the integrity of the photosynthesis system. Additionally, transgenic plants had high proline content and relatively lower ROS levels under drought and salinity stress conditions.

Collectively, *PdMT2A* overexpression improved stress tolerance in transgenic *Arabidopsis* plants by maintaining chlorophyll, high K^+^/Na^+^ ratio, high proline content and decreased ROS levels under drought and salinity stresses. Overall, this study represents one of the very few reports that functionally characterized stress-responsive genes from the date palm.

## Supplementary Materials

Supplementary materials can be found at https://www.mdpi.com/1422-0067/20/12/2871/s1.
