# Supplementary material for: Overexpression of a Metallothionein 2A Gene from Date Palm Confers Abiotic Stress Tolerance to Yeast and Arabidopsis thaliana
Source: Int J Mol Sci. 2019 Jun 12;20(12):2871. doi: 10.3390/ijms20122871 (PMC6627811; doi:10.3390/ijms20122871)
Supplement: Supplementary file 1 [file ijms-20-02871-s001.pdf]

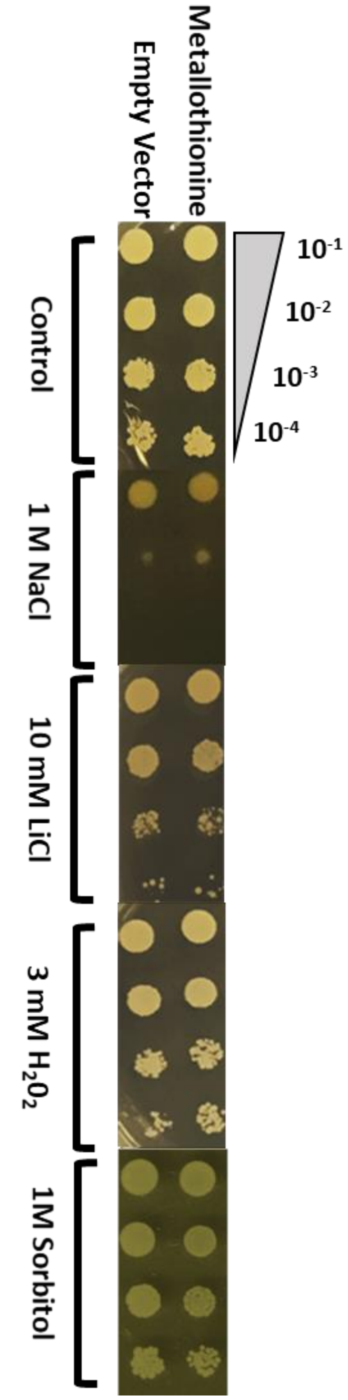

Supplementary Figure S1. Overexpression of *PdMT2A* in INVSc wild type yeast strain. The effect of *PdMT2A* transgene on the growth of wild type yeast cells, the relative tolerance of TY and EV cells tested by yeast spot assay when grown under control and other different abiotic stress conditions on solid media

Supplementary Table S1. List of oligos used for qPCR analysis.

Genes	Primers
AtCHX20-F	TGCGCGTATTTTAGCAGAGC
AtCHX20-R	ATCGTTAAAAGCGGCTGCAG
AtSOS1-F	TCGTTTCAGCCAAATCAGAAAGT
AtSOS1-R	CGCATGTTTACGGGTTTCAA
AtHKT1-F	CAATCACCGAAAGGCAAAAT
AtHKT1-R	CGTCCTGCAAACCCATAACT
AtVacuolar Na+/H+ Antiporter-F	ATTGAGCCTTCAGGGAACCA
AtVacuolar Na+/H+ Antiporter-R	CCGTGTCAAGAAGCCACGTATA
AtABA stress induced gene - F	GCTGGGCCTATCGTGATGTT
AtABA stress induced gene - R	TCCAATACGCAAGCCACTGTT
AtSOD-F	TGCCACCTTCACAATCACTG
AtSOD-R	TCTGCATGGACAACAACAGC
AtActin2-F	TCCCTCAGCACATTCCAGCAGAT
AtActin2-R	AACGATTCCTGGACCTGCCTCATC
